# Personal

**Published:** 1867-10

**Authors:** 


### Personal.

A letter just received from Prof. Freer, announces that he will take the steamer "home again" on the 12th inst. A racy and instructive letter from his pen will appear in the next No. of the JOURNAL. His health is thoroughly reëstablished, and his friends will be delighted to greet him *in propria persona.*

